# Exploring the Mechanical Anisotropy and Ideal Strengths of Tetragonal B_4_CO_4_

**DOI:** 10.3390/ma10020128

**Published:** 2017-02-04

**Authors:** Baobing Zheng, Meiguang Zhang, Canjun Wang

**Affiliations:** College of Physics and Optoelectronics Technology, Nonlinear Research Institute, Baoji University of Arts and Sciences, Baoji 721016, China; zhmgbj@126.com (M.Z.); cjwangbj@126.com (C.W.)

**Keywords:** anisotropic properties, ideal strengths, superhard, B-C-O compound

## Abstract

First-principles calculations were employed to study the mechanical properties for the recently proposed tetragonal B_4_CO_4_ (*t*-B_4_CO_4_). The calculated structural parameters and elastic constants of *t*-B_4_CO_4_ are in excellent agreement with the previous results, indicating the reliability of the present calculations. The directional dependences of the Young’s modulus and shear modulus for *t*-B_4_CO_4_ are deduced in detail, and the corresponding results suggest that the *t*-B_4_CO_4_ possesses a high degree of anisotropy. Based on the strain-stress method, the ideal tensile and shear strengths along the principal crystal directions are calculated, and the obtained results indicate that the shear mode along (001)[100] slip system dominates the plastic deformation of *t*-B_4_CO_4_, which can be ascribed to the breaking of the ionic B-O bonds. The weakest ideal shear strength of 27.5 GPa demonstrates that the *t*-B_4_CO_4_ compound is not a superhard material, but is indeed a hard material. Based on the atomic explanation that the ternary B-C-O compounds cannot acquire high ideal strength, we propose two possible routes to design superhard B-C-O compounds.

## 1. Introduction

Superhard materials, defined as materials with Vickers hardness higher than 40 GPa, are of great interest in many industrial areas, such as abrasives, polishing, cutting tools, and protective coatings. Generally, superhard materials require a high valence electron density and high bond covalency to form strong covalent bonds that would enhance their structural strength against large inelastic deformations, consequently leading to increased hardness. Therefore, the recent quest for intrinsic superhard materials mainly concentrates on two categories of compounds [1]. One category consists of the compounds formed by electron-rich transition-metal (TM) and small first row main group elements (e.g., boron, carbon, and nitrogen), such as ReB_2_ [2], OsB_2_ [3], WB_4_ [4,5], FeB_4_ [6], Os_2_C [7], PtN_2_ [8], IrN_2_ [9], etc. Although these compounds exhibit large bulk moduli close to that of diamond and are hence considered as ultra-incompressible materials, further experimental and theoretical studies suggest that most of the TM borides, carbide, and nitrides are unlikely to be superhard [7,10,11,12,13]. The other category consists of light element compounds (B-C-N-O) with strong covalent bonds, such as B-C compounds (BC_3_ [14], BC_5_ [15]), B-C-N compounds (BC_2_N [16]), B-O compounds (B_6_O [17]), and B-C-O compounds.

In particular, the successful syntheses of the B-C-O system have spurred extensive research efforts in the study of those compounds due to their excellent mechanical properties [18,19,20]. However, the stoichiometric ratios of recently synthesized B-C-O systems under high pressures, which belong to the single crystal of the interstitial phases based on the rhombohedral *α*-boron modification, are very complex. Garvie et al. [18] prepared a series of ternary phases in the B-C-O system with the mixtures of B, C, and B_2_O_3_ under pressure between 5 and 7.5 GPa and a temperature of 1700 °C. The boron suboxycarbide B(C,O)_0.155_ single crystal has been successfully synthesized by chemical reaction between the B_4_C and B_2_O_3_ compounds under a pressure of 5.5 GPa and a temperature of 1400 K [19]. Theoretically, the advent of density functional theory has provided us a powerful tool for theoretical studies when treating the energetics/structure/bonding of carbon/boron-based systems [7,12,13,21,22,23]. Moreover, the vigorous development of crystal structure prediction methods makes it possible to efficiently design functional materials that only require the chemical compositions. Based on the particle swarm optimization technique, the potential superhard compound of BC_2_O in the B-C-O system is proposed with theoretical Vickers hardness of 50 GPa [24]. Recently, Liu et al. predicted a lonsdaleite-like orthorhombic structure B_2_CO with strong *sp*^3^ covalent B-C and B-O bonds [25]. Very recently, we studied the higher carbon content in ternary B_2_C_x_O compounds which are isoelectronic with diamond, in order to search for novel superhard materials [26]. However, the three ultra-incompressible and thermodynamically stable B_2_C_x_O compounds possess substantially lower ideal shear strengths than those of diamond and *c*-BN, suggesting that they may not be intrinsically superhard in spite of the calculated hardness being higher than 40 GPa. Using the evolutionary algorithm, Wang et al. [27] explored a new tetragonal thermodynamically stable phase B_4_CO_4_ (space group $I\bar{4}$) with a claimed hardness of ~40 GPa, indicating that the tetragonal B_4_CO_4_ (*t*-B_4_CO_4_) is potentially superhard. Intuitively speaking, despite its tetrahedrally coordinated B and C atoms, the presence of weak B-O bonds and the large hollow space in the center of the *t*-B_4_CO_4_ structure suggest that whether or not *t*-B_4_CO_4_ is a superhard material needs to be further clarified.

In the present work, using the first-principles calculations, we systematically investigated the mechanical properties of the *t*-B_4_CO_4_ in comparison with other B-C-O structures. The formulas of the Young’s and shear moduli along the arbitrary directions for Laue class 4/*m* of the tetragonal crystal system were deduced, in order to study their elastic anisotropy. The ideal strengths were estimated to clarify whether the *t*-B_4_CO_4_ is superhard and to provide an atomic explanation of its plastic deformation.

## 2. Computational Methods

Using density functional theory with the Perdew-Burke-Ernzerhof (PBE) exchange correlation, we carried out the geometry optimization and the total energy calculations as implemented in the Vienna ab initio Simulation Package (VASP) [28,29]. The projector augmented-wave (PAW) method combined with the frozen core approximation was employed to describe the electron and core interactions [30], where the 2*s*^2^2*p*^1^, 2*s*^2^2*p*^2^, and 2*s*^2^2*p*^4^ are considered as valence electrons for B, C, and O, respectively. The total-energy and elastic constants were calculated with a 550 eV plane-wave cutoff energy and a grid of 0.03 Å^−1^ Monkhorst-Pack *k* point meshes [31], which are accurate enough to ensure that the enthalpy results were well converged to below 1 meV/f.u. The successfully utilized strain-stress method was introduced to evaluate the elastic constants. The bulk modulus, shear modulus, and Young’s modulus were calculated via the Voigt-Reuss-Hill approximation [32]. We applied continuous deformation upon the *t*-B_4_CO_4_ cell by increasing the displacement in the direction of the corresponding strain, and then estimated the ideal tensile and shear strengths from the yield stress [33,34]. The convergence tests indicate that the calculated elastic constants and ideal strengths are almost independent of the energy cutoff, *k*-point mesh, and smearing parameter (see Supplemental Material).

## 3. Results and Discussion

**Figure 1:**
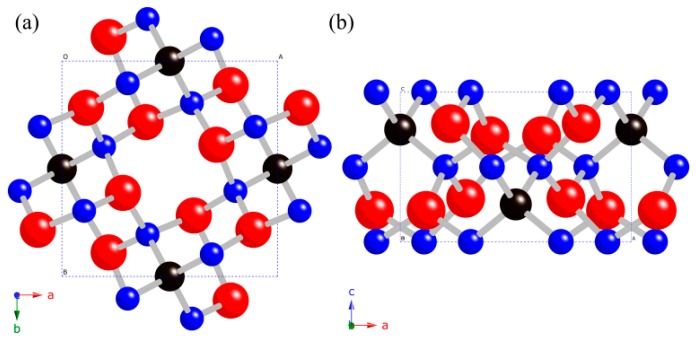
Crystal structures of *t*-B_4_CO_4_ as viewed along the [001] direction (**a**) and the [010] direction (**b**), the red, blue, and black spheres represent O, B, and C atoms, respectively.

The optimized structures of *t*-B_4_CO_4_ along the [001] and [010] view directions are shown in Figure 1. Each C atom is coordinated with four B atoms, and each B atom is coordinated with one C atom and three O atoms. All the lengths of the B-C bonds are 1.571 Å, and the bond lengths of the three kinds of B-O bonds are 1.505 Å, 1.543 Å, 1.569 Å, respectively, which is consistent with the previous results of the average B-C and B-O bond lengths of 1.570 Å and 1.539 Å at ambient pressure [27]. The slight differences between our estimated values and previous results suggest that the present calculations are accurate and reliable. The calculated elastic constants *C_ij_*, bulk modulus *B*, shear modulus *G*, and Young’s modulus *G* at ambient pressure are summarized in Table 1 together with other B-C-O systems for comparison.

For a tetragonal $I\bar{4}$ crystal, the seven *C_ij_* should satisfy the following necessary and sufficient stability conditions according to the Born stability criterion [38]:

(1) $$\begin{array}{l} C_{11}>\left|C_{12}\right|,\;2C_{13}^{2}<C_{33}\left(C_{11}+C_{12}\right),\\ C_{44}>0,\;2C_{16}^{2}<C_{66}\left(C_{11}-C_{12}\right). \end{array}$$

Clearly, the calculated elastic constants of *t*-B_4_CO_4_ meet all the mechanical stability criteria, indicating the mechanical stability of *t*-B_4_CO_4_. Compared with the other B-C-O systems from Table 1, we find that the *C*_11_ and *C*_33_ of *t*-B_4_CO_4_ are significantly less than those of B_2_CO, B_2_C_2_O, B_2_C_3_O, and B_2_C_5_O, as well as the superhard *c*-BN and diamond, suggesting its lower incompressibility along the *a*- and *c*-direction. The bulk modulus (248 GPa) and shear modulus (218 GPa) of *t*-B_4_CO_4_ are not only much smaller than those of typical superhard *c*-BN and diamond, but are also much smaller than those of the ternary B-C-O system. Although the plastic hardness is essentially inequivalent to the elastic modulus, the value of the elastic modulus indirectly reflects the hardness of the material. Thus, the noticeable difference of elastic moduli between *t*-B_4_CO_4_ and superhard *c*-BN or diamond suggest that the superhard feature of *t*-B_4_CO_4_ should be further debated.

Anisotropy is a measure of a material's directional dependence of its physical or mechanical properties. Although all the known crystals are elastically anisotropic due to the atomic arrangement of the crystal structure of the material, the elastic anisotropy of crystal can actually play a dominant role in plastic deformation, crack behavior, and elastic instability when the anisotropy of a single crystal is large and crucial. Therefore, we systematically investigated the elastic anisotropy of the *t*-B_4_CO_4_ compound for its potential engineering applications. For a tetragonal $I\bar{4}$ structure, the Young’s modulus for tensile stress along an arbitrary [*hkl*] direction can be expressed as the following equation:

(2) $$\begin{array}{l} E^{-1}=s_{11}(\alpha^{4}+\beta^{4})+s_{33}\gamma^{4}+2s_{12}\alpha^{2}\beta^{2}+2s_{13}(\beta^{2}\gamma^{2}+\alpha^{2}\gamma^{2})\\ +s_{44}(\beta^{2}\gamma^{2}+\alpha^{2}\gamma^{2})+s_{66}\alpha^{2}\beta^{2}+2s_{16}\alpha \beta (\alpha^{2}-\beta^{2}) \end{array}$$

where *α*, *β*, and *γ* are the direction cosines of the tensile stress direction deduced from the transformed coordinate system with respect to the original coordinate system, and *s*_11_, *s*_12_, *s*_13_, *s*_33_, *s*_44_, *s*_66_, and *s*_16_ are the independent elastic compliance constants given by Kelly et al. [39], which are determined from the calculated elastic constants *C_ij_*. For the Laue class 4/*m* of the tetragonal crystal system, the extra elastic constant *C*_16_ brings the total number of independent elastic compliances *s_ij_* to seven:

(3) $$\begin{array}{l} s_{11}=s_{22}=\frac{1}{2}\left(\frac{C_{33}}{C^{\prime }}+\frac{C_{66}}{C^{\prime \prime }}\right),\;s_{12}=\frac{1}{2}\left(\frac{C_{33}}{C^{\prime }}-\frac{C_{66}}{C^{\prime \prime }}\right),\;s_{13}=-\frac{C_{13}}{C^{\prime }},\;s_{33}=\frac{C_{11}+C_{12}}{C^{\prime }}\\ s_{44}=\frac{1}{C_{44}},\;s_{66}=\frac{C_{11}-C_{12}}{C^{\prime \prime }},\;s_{16}=-\frac{C_{16}}{C^{\prime \prime }} \end{array}$$

where

(4) $$C^{\prime }=C_{33}\left(C_{11}+C_{12}\right)-2C_{13}^{2},\;C^{\prime \prime }=C_{66}\left(C_{11}-C_{12}\right)-2C_{16}^{2}$$

The analytical formulas of the Young’s moduli for the tensile axis within specific planes, such as the (001), (100), and $\left(1\bar{1}0\right)$ tensile planes, are deduced from Equation (2) and are then listed in Table 2, where *θ* is the angle between the principal crystal direction of the tensile plane and the tensile stress direction.

**Figure 2:**
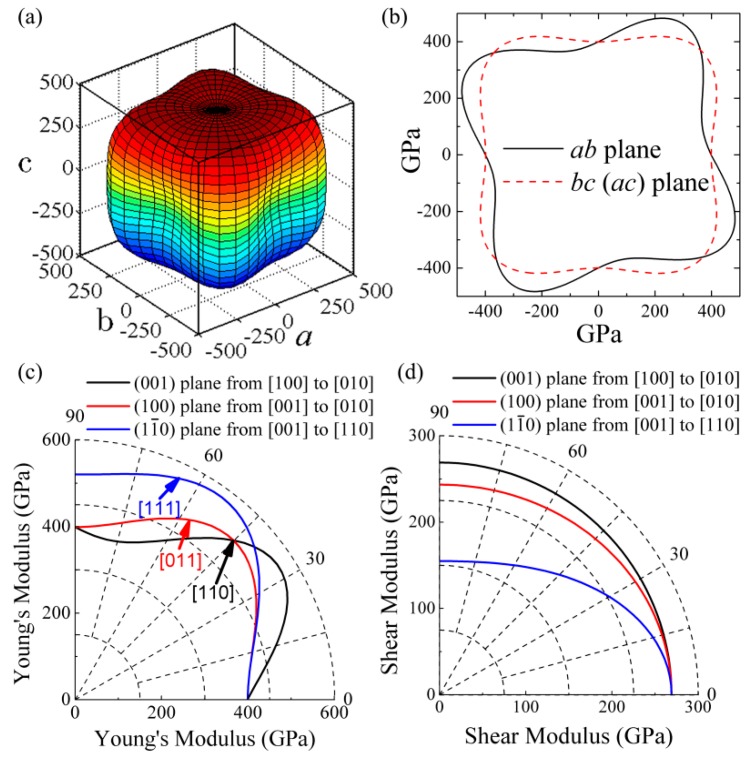
Orientation dependence of Young’s Modulus *E* (**a**,**c**) and the corresponding projection in the *ab*, *ac*, and *bc* planes (**b**) for the *t*-B_4_CO_4_, orientation dependence of the shear modulus of *t*-B_4_CO_4_ (**d**).

The three-dimensional (3D) surface representation, the three-plane projection drawings, and the orientation dependences in polar coordinates of the Young’s modulus *E* are plotted in Figure 2a–c, based on the analytical formulas in Table 2, respectively. The shape of the 3D surface representation far from sphere suggests that the Young’s modulus of *t*-B_4_CO_4_ possesses a high degree of anisotropy. The projection drawing of the *ac* and *bc* planes for the 3D surface representation coincide with each other due to the symmetry of *t*-B_4_CO_4_. Interestingly, unlike the Laue class 4/*mmm* of the tetragonal crystal, the extra elastic constant *C*_16_ of *t*-B_4_CO_4_ tilts the projection graph of the *ab* plane. From Figure 2c, we can conclude that the sequence of Young’s moduli along the principal crystal directions is as follows: *E*_[010]_ < *E*_[001]_ < *E*_[011]_ < *E*_[110]_ < *E*_[111]_. Note that the maximum Young’s modulus in the principal crystal directions appears in the [111] direction, which shows a satisfactory agreement with the fact that the covalent B-C bonds with high bond strength for *t*-B_4_CO_4_ mainly distribute in the [111] direction.

The shear modulus *G* on the (*hkl*) shear plane with shear stress applied along the [*uvw*] direction is given by

(5) $$\begin{array}{l} G^{-1}=4s_{11}\left(\alpha_{1}^{2}\alpha_{2}^{2}+\beta_{1}^{2}\beta_{2}^{2}\right)+4s_{33}\gamma_{1}^{2}\gamma_{2}^{2}+8s_{12}\alpha_{1}\alpha_{2}\beta_{1}\beta_{2}+s_{66}{\left(\alpha_{1}\beta_{2}+\alpha_{2}\beta_{1}\right)}^{2}\\ +8s_{13}\left(\beta_{1}\beta_{2}\gamma_{1}\gamma_{2}+\alpha_{1}\alpha_{2}\gamma_{1}\gamma_{2}\right)+s_{44}\left[{\left(\beta_{1}\gamma_{2}+\beta_{2}\gamma_{1}\right)}^{2}+{\left(\alpha_{1}\gamma_{2}+\alpha_{2}\gamma_{1}\right)}^{2}\right]\\ +4s_{16}\left(\alpha_{1}\beta_{2}+\beta_{1}\alpha_{2}\right)\left(\alpha_{1}\alpha_{2}-\beta_{1}\beta_{2}\right) \end{array}$$

where (*α*_1_, *β*_1_, *γ*_1_) and (*α*_2_, *β*_2_, *γ*_2_) are the direction cosines of the [*uvw*] and [*HKL*] directions in the primitive coordinate system, respectively, and the [*HKL*] directions denote the vector normal to the (*hkl*) shear plane. For a given shear plane, Equation (5) can be further simplified according to the orientation angle *θ* between the shear stress direction and the specified crystal direction. The deduced formulas of shear moduli along the (001), (100), and $\left(1\bar{1}0\right)$ shear planes are summarized in Table 3. The orientation dependences of the shear modulus of *t*-B_4_CO_4_ are hence plotted in Figure 2d for the shear (001), (100), and $\left(1\bar{1}0\right)$ planes. It is clear that the shear modulus within the (001) basal plane is independent of the orientation angle *θ*, which results from the fact that the analytical formula of the shear modulus within the (001) basal plane is G(001)=1s44=C44=269 GPa. On the other hand, the shear moduli within the (100) and $\left(1\bar{1}0\right)$ basal planes gradually decrease with the increase of the orientation angle *θ*.

The hardness of *t*-B_4_CO_4_ estimated by the Lyakhov-Oganov model and the Chen-Niu model are 39 GPa and 38 GPa [24], respectively, which are in excellent agreement with each other, and are both close to 40 GPa. The Lyakhov-Oganov model for the Knoop hardness can be described by the following formula [40]:

(6) $$H_{k}(\mathrm{GPa})=\frac{423.8}{V}n{\left[\prod_{k=1}^{n}N_{k}X_{k}e^{-2.7f_{k}}\right]}^{1/n}-3.4,$$

where *N_k_* is the number of bonds of type *k* in the unit cell, and *X_k_* and *f_k_* are the electron-holding energy of the bond *k* relevant to the electronegativities of atoms and its ionicity indicator, respectively. The Chen-Niu model can be summarized as follows [41]:

(7) $$H_{V}(\mathrm{GPa})=2{\left(k^{2}G\right)}^{0.585}-3,\;k=G/B.$$

Clearly, the two models based on either the elastic moduli or the electronegativity and covalent radii are actually not appropriate to describe the plastic hardness of a material. Physically, the indentation hardness in an experiment is measured under the condition of fully developed plasticity [42], and the plastic deformation of materials usually occurs far from equilibrium. However, all the introduced parameters in the two models are obtained under the equilibrium structure, which suggests that we should further check the superhard feature of *t*-B_4_CO_4_ employing other appropriate criteria. The ideal strengths, in particular the ideal shear strength, describe the resistance of the system at the atomic level where plastic deformation occurs, and thus are more suitable for assessing the possibility that a material may be superhard [42].

**Figure 3:**
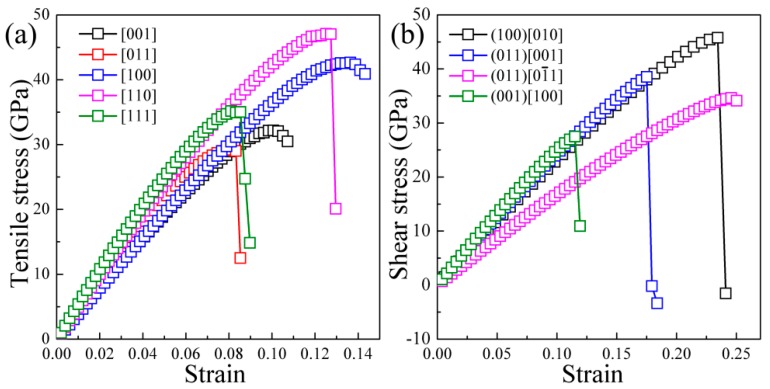
Calculated stress-strain relations for *t*-B_4_CO_4_ in various tensile (**a**) and shear (**b**) directions.

Here, we deform the *t*-B_4_CO_4_ cell gradually in the direction of the applied strain and then obtain the ideal strengths when the cell becomes mechanically unstable. Figure 3a,b illustrates the calculated strain-stress relations under tensile and shear stress along the specified directions, respectively. The estimated tensile strengths in the [001], [011], [100], [110], and [111] directions are 32.2, 29.5, 42.6, 47.1, and 35.0 GPa, respectively. As listed in Table 1, the weakest tensile strengths of other B-C-O compounds except tP4-B_2_CO are quantitatively comparable to that of *t*-B_4_CO_4_, which occurs in the [011] tensile direction. Compared to diamond (82.3 GPa) and *c*-BN (55.3 GPa), the minimum tensile strength of *t*-B_4_CO_4_ (29.5 GPa) is not only lower than that of superhard diamond but is also lower than superhard *c*-BN. Moreover, the largest tensile strength of *t*-B_4_CO_4_ along the principal crystal direction is only 47.1 GPa, indicating the weak ability of the tensile resistance and the tendency to fracture under relatively small tensile stress for *t*-B_4_CO_4_.

Generally, plastic deformation occurs in shear, so the ideal shear strength is more suitable for measuring whether a material may be superhard rather than theoretical hardness calculated by semi-empirical or first-principles methods. The lowest ideal shear strength of 27.5 GPa is found along the (001)[100] slip system for *t*-B_4_CO_4_. This value is markedly lower than the minimum shear strengths of *c*-BN (58.3 GPa) and diamond (86.8 GPa), but higher than those of other B-C-O compounds (see Table 1). The value of 27.5 GPa is much less than the superhard criterion of 40 GPa, and suggests that the *t*-B_4_CO_4_ compound is therefore intrinsically hard, but not superhard as we expected. Note that the minimum shear strength is smaller than the minimum tensile strength. Thus, the shear mode in the (001)[100] slip system dominates the plastic deformation of *t*-B_4_CO_4_ rather than the tensile mode.

**Figure 4:**
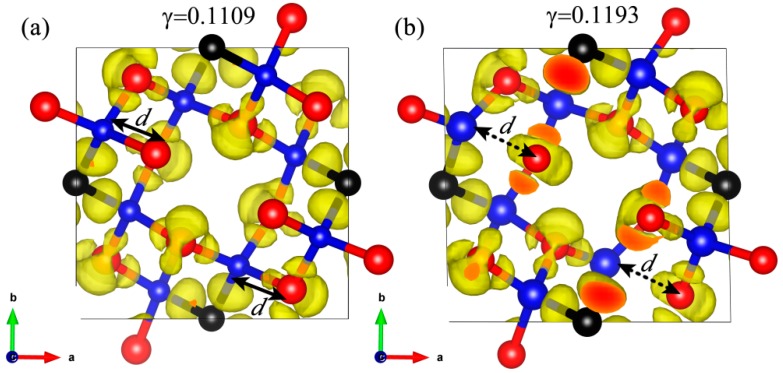
Structural and ELF transformation before (**a**) and after (**b**) the lattice instability for *t*-B_4_CO_4_.

Figure 3b shows a sharp decrease at a critical strain of *γ* = 0.1151 along the (001)[100] slip system, implying a lattice instability for *t*-B_4_CO_4_. We next explored the atomic explanation for the plastic structural deformation of *t*-B_4_CO_4_ along the (001)[100] slip system. The Bader charge analysis revealed that the numbers of Bader charges for the B and O atoms are 2.80*e* and 9.57*e* in *t*-B_4_CO_4_, respectively, suggesting the ionic feature of B-O bonds. Furthermore, as plotted in Figure 4, the isosurface of the electron localization function (ELF) for *t*-B_4_CO_4_ shows the valence electrons of B-O bonds are highly localized around the O atoms, which further confirms the ionic feature of B-O bonds. Figure 4 also illustrates the structural transformation of the *t*-B_4_CO_4_ before and after the lattice instability, i.e., at a shear strain of *γ* = 0.1109 and *γ* = 0.1193. Clearly, although the B-C bonds in *t*-B_4_CO_4_ do not present significant changes, the lengths of part of the B-O bonds (denoted as double-arrow lines) increase visibly from 1.724 Å to 2.364 Å before and after the lattice instability. It is well known that the strength of an ionic bond is much weaker than that of a covalent bond, which indicates the lattice instability of *t*-B_4_CO_4_ may result from the breaking of B-O bonds rather than B-C bonds. A bond critical point (BCP) analysis revealed that the charge density at the B-O bond critical point decreases from 0.624 electrons/Å^3^ to 0.183 electrons/Å^3^ when the shear strain increases from *γ* = 0.1109 to *γ* = 0.1193. Meanwhile, the Laplacian value decreases significantly from 2.973 to 1.231. All these results support the fact that the lattice instability of *t*-B_4_CO_4_ under shear load originates from the breaking of B-O bonds.

**Figure 5:**
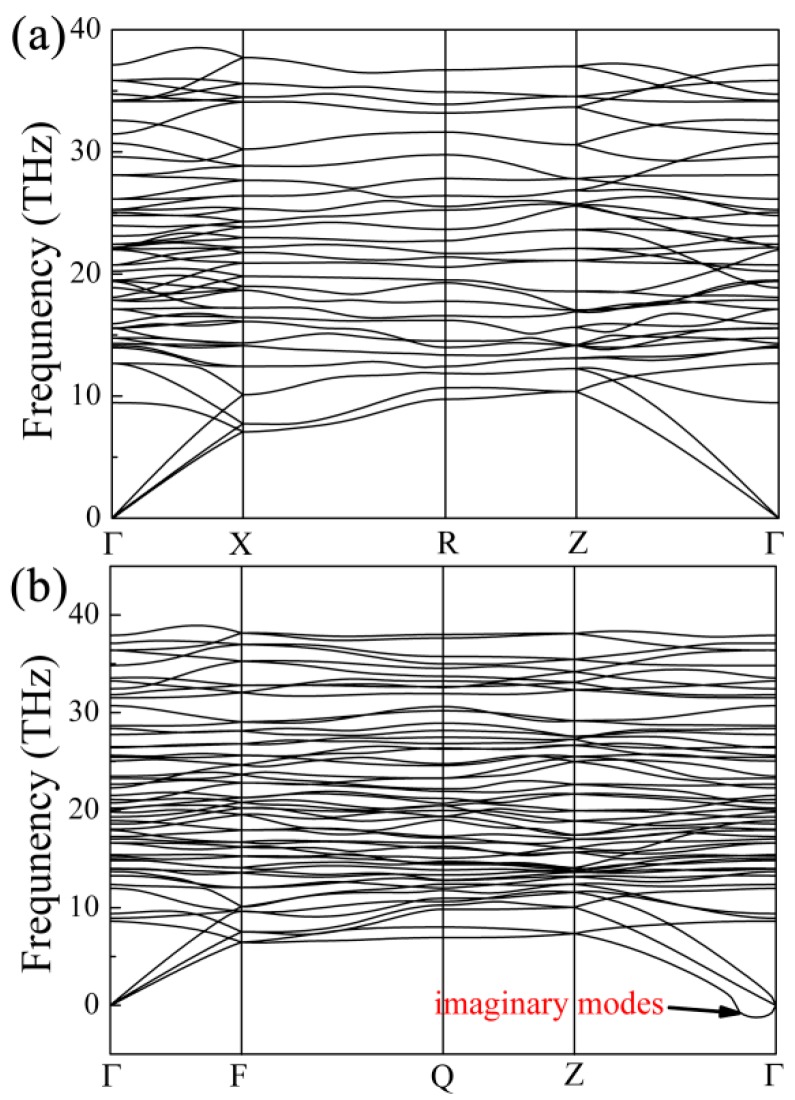
Calculated phonon dispersion curves for *t*-B_4_CO_4_ before (**a**) and after (**b**) shear deformation.

It is well known that the lattice instability of the material is directly relevant to the imaginary modes of phonon dispersion of this material. To really understand the origin of the lattice instability and the key mechanisms that dominate the shear deformation, we calculated the phonon dispersion of *t*-B_4_CO_4_ at the shear strain of *γ* = 0.1193 in the (001)[100] slip system. The obtained dispersion curves by the finite displacement method before and after shear deformation are plotted in Figure 5. Clearly, imaginary frequencies are found near the Г point, and the most unstable modes occur at the (0, 0, 0.05) point along the Г to Z direction, which suggests that the lattice of *t*-B_4_CO_4_ has become unstable at the large shear deformation. Further analysis of the eigenvectors for the largest imaginary phonon modes indicates that the atoms of *t*-B_4_CO_4_ mainly vibrate along the *a* direction, and the vibrations along the *b* and *c* directions are very slight. This result can be attributed to the fact that we apply the shear strain along the *a* direction in the ideal strength calculations. Meanwhile, due to the displacement of B and O atoms along the *a* direction at sufficiently large deformation, the B-O bonds are hence broken, leading to the collapse of the *t*-B_4_CO_4_ lattice.

Note that the minimum ideal shear strength of *t*-B_4_CO_4_ can only reach 27.5 GPa, which is much lower than the superhard criterion of 40 GPa, as well as the other ternary B-C-O compounds (See Table 1), suggesting that all these ternary B-C-O compounds currently known may not be intrinsically superhard in spite of their relatively high elastic moduli. The atomic explanation that the ternary B-C-O compounds cannot acquire higher hardness is completely due to the weak B-O bonds being easy to break without exception. Thus, we believe that the possible routes to design the superhard B-C-O compounds can be summarized as follows: (1) one is to reduce the proportion of weak B-O ionic bonds and increase the proportion of strong B-C covalent bonds as much as possible in ternary B-C-O compounds; (2) the other is to design special polyhedral crystal structures with less multi-center bridge bonds to resist the shear strain, such as B_12_ icosahedra in B_6_O.

## 4. Conclusions

In summary, we have systematically investigated the mechanical properties of *t*-B_4_CO_4_, including the elastic constants, bulk modulus, Young’s modulus, and shear modulus, in comparison with other ternary B-C-O compounds. The elastic anisotropies of *t*-B_4_CO_4_ are illustrated according to the analytical formulas of the Young’s and shear moduli along different crystal orientations. To examine the superhard feature of *t*-B_4_CO_4_, the ideal strengths of *t*-B_4_CO_4_ are estimated based on the strain-stress method. The dominating ideal strength of 27.5 GPa is found along the (001)[100] slip system, indicating that the structural collapse of *t*-B_4_CO_4_ is far from 40 GPa. Thus, we can conclude that *t*-B_4_CO_4_ is not intrinsically superhard, but is indeed a hard material. We expect that the present work can establish an appropriate approach to distinguish whether a material is superhard, and provide new routes to design the novel superhard B-C-O compounds.

## Figures and Tables

**Table 1:** Calculated Elastic Constants *C_ij_*, Bulk Modulus *B*, Shear Modulus *G*, Young’s Modulus *E*, and ideal strength (minimum tensile strength ***σ_min_*** and shear strength ***τ_min_***) of *t*-B_4_CO_4_ together with other ternary B-C-O compounds (in units of GPa).

Compounds	Source	*C*_11_	*C*_33_	*C*_44_	*C*_66_	*C*_12_	*C*_13_	*C*_16_	*B*	*G*	*E*	*σ_min_*	*τ_min_*
*t*-B_4_CO_4_	This work	481	452	269	260	150	129	−52	248	218	505	$\sigma_{\left[011\right]}$ = 29.0	$\tau_{\left(001)[100\right]}$ = 27.5
	Theory ^1^	480	449	268	259	152	131		248	220	509		
tI16-B_2_CO	Theory ^2^	600	646	304	283	182	144		310	265			
tP4-B_2_CO	Theory ^3^	736	591	240	254	53	157		311	254		$\sigma_{\left[111\right]}$ = 6.1	$\tau_{\left(111)[1\bar{1}0\right]}$ = 2.6
oP8-B_2_CO	Theory ^4^	732	675	238	260	112	69		298	270			
B_2_C_2_O	Theory ^5^	763	590	229	174	15	135		299	264	611	$\sigma_{\left[111\right]}$ = 21.3	$\tau_{\left(111)[1\bar{1}0\right]}$ = 11.1
B_2_C_3_O	Theory ^5^	808	664	283	299	32	183		322	302	690	$\sigma_{\left[111\right]}$ = 22.4	$\tau_{\left(111)[1\bar{1}0\right]}$ = 11.4
B_2_C_5_O	Theory ^5^	889	740	346	335	30	135		345	351	787	$\sigma_{\left[111\right]}$ = 25.1	$\tau_{\left(111)[1\bar{1}0\right]}$ = 16.3
B_2_O	Theory ^2^	327	497	232	207	230	144		242	124			
*c*-BN	Theory ^6^	786		445		172			376	390		$\sigma_{\left[111\right]}$ = 55.3	$\tau_{\left(111)[11\bar{2}\right]}$ = 58.3
Diamond	Theory ^7^	1052		555		122			432	517	1109	$\sigma_{\left[111\right]}$ = 82.3	$\tau_{\left(111)[11\bar{2}\right]}$ = 86.8

^1^ Ref. [27]; ^2^ Ref. [24]; ^3^ Ref. [24,26]; ^4^ Ref. [25]; ^5^ Ref. [26]; ^6^ Ref. [35]; ^7^ Ref. [36,37].

**Table 2:** Formulas of Young’s moduli for the tensile axis within specific planes.

Tensile Plane	$E^{-1}$	Orientation Angle *θ*
(001)	$s_{11}-\frac{1}{4}(2s_{11}-2s_{12}-s_{66})\sin^{2}2\theta +s_{16}\sin2\theta (\sin^{2}\theta -\cos^{2}\theta )$	between [*hk*0] and [100]
(100)	$s_{11}\sin^{4}\theta +s_{33}\cos^{4}\theta +\frac{1}{4}(2s_{13}+s_{44})\sin^{2}2\theta$	between [0*kl*] and [001]
$\left(1\bar{1}0\right)$	$\frac{1}{4}(2s_{11}+2s_{12}+s_{66})\sin^{4}\theta +s_{33}\cos^{4}\theta +\frac{1}{4}(2s_{13}+s_{44})\sin^{2}2\theta$	between [hkl] and [001]

**Table 3:** Formulas of shear moduli for the shear stress direction within specific planes.

Shear Plane	$G^{-1}$	Orientation Angle *θ*
(001)	$s_{44}$	between [*uvw*] and [100]
(100)	$s_{66}+(s_{44}-s_{66})\cos^{2}\theta$	between [*uvw*] and [001]
$\left(1\bar{1}0\right)$	$2(s_{11}-s_{12})\sin^{2}\theta +s_{44}\cos^{2}\theta$	between [*uvw*] and [001]

## Supplementary Materials

The following are available online at www.mdpi.com/1996-1944/10/2/128/s1. Table S1: The convergence tests of exchange-correlation functional, Table S2: The convergence tests of cutoff energy and *k*-point mesh, Table S3: The convergence tests of the width of the smearing, Table S4: The convergence tests of cutoff energy and *k*-point mesh for ideal strength calculations.
