# Supplementary material for: Exploring the Mechanical Anisotropy and Ideal Strengths of Tetragonal B4CO4
Source: Materials (Basel). 2017 Feb 4;10(2):128. doi: 10.3390/ma10020128 (PMC5459106; doi:10.3390/ma10020128)
Supplement: Supplementary file 1 [file materials-10-00128-s001.pdf]

Supplementary Materials: Exploring the Mechanical Anisotropy and Ideal Strengths of Tetragonal B_4CO_4

Baobing Zheng, Meiguang Zhang and Canjun Wang

It is well known that the exchange-correlation functional, energy cutoff, *k*-point mesh as well as the smearing parameter etc. have a great impact on the elastic constants. Thus, it is necessary to estimate the computational error introduced by these parameters when we perform the DFT calculations. Generally, the frequently-used exchange-correlation functional can be divided as two types: generalized gradient approximation (GGA) and local density approximation (LDA). Here we mainly show the errors introduced by these two kinds of exchange-correlation functional, the corresponding results are listed in Table S1. It is clear that the elastic constants, bulk modulus, shear modulus, and Young's modulus obtained by LDA are higher than those of GGA, which can be ascribe to the well-known reason that LDA usually underestimates the lattice parameters and hence overestimates the elastic constants and moduli. Moreover, the GGA applied in our present work is the most popular and effective approximation to calculate the elastic constants for superhard materials, and has been successfully applied in numerous previous works [1–7] when studying the system containing B, C, and O. Thus, we here adopt GGA exchange-correlation functional.

Table S1. The convergence tests of exchange-correlation functional.

Exchange-Correlation Functional	*k*-Point Mesh	C_{11}	C_{33}	C_{44}	C_{66}	C_{12}	C_{13}	C_{16}	B	G	E
GGA	5 × 5 × 8	480.7	452.5	268.7	260.3	149.9	129.2	−52.7	247.6	217.6	504.9
	6 × 6 × 9	481.0	451.7	268.6	260.2	149.5	129.2	−52.7	247.5	217.6	504.9
	7 × 7 × 11	480.8	452.4	268.7	260.3	149.9	129.3	−52.7	247.6	217.6	505.0
LDA	5 × 5 × 8	514.6	482.4	293.2	277.7	166.4	148.7	−60.0	271.2	231.7	540.9
	6 × 6 × 9	514.6	482.9	293.0	277.5	166.6	148.6	−60.1	271.3	231.6	541.0
	7 × 7 × 11	514.4	482.6	292.6	277.6	166.6	148.5	−60.1	271.2	231.6	540.9

We recalculated the single crystal elastic constants using different cutoff energy, *k*-point mesh, and smearing parameter, and the obtained results are summarized in Tables S2 and S3. Clearly, the elastic constants and moduli calculated by the higher criteria are almost independent with the cutoff energy, *k*-point mesh and the smearing parameter, suggesting the accuracy and reliability of our calculations.

Table S2. The convergence tests of cutoff energy and *k*-point mesh.

Cutoff Energy	*k*-Point Mesh	C_{11}	C_{33}	C_{44}	C_{66}	C_{12}	C_{13}	C_{16}	B	G	E
550 eV	5 × 5 × 8	480.7	452.5	268.7	260.3	149.9	129.2	−52.7	247.6	217.6	504.9
	6 × 6 × 9	481.0	451.7	268.6	260.2	149.5	129.2	−52.7	247.5	217.6	504.9
	7 × 7 × 11	480.8	452.4	268.7	260.3	149.9	129.3	−52.7	247.6	217.6	505.0
650 eV	5 × 5 × 8	484.7	454.3	271.5	262.6	150.1	131.6	−53.4	249.8	219.2	508.7
	6 × 6 × 9	487.3	454.4	272.2	262.7	150.2	133.6	−54.5	248.3	221.9	512.9
	7 × 7 × 11	484.2	454.3	272.3	262.6	149.1	130.1	−53.4	248.6	2196	508.9
800 eV	5 × 5 × 8	480.8	452.2	268.7	260.4	150.1	129.1	−52.7	247.6	217.7	505.0
	6 × 6 × 9	482.8	452.8	269.1	261.2	148.7	129.2	−53.0	247.6	218.3	506.1
	7 × 7 × 11	480.7	451.9	268.6	260.2	149.9	129.0	−52.7	247.5	217.6	504.8

Table S3. The convergence tests of the width of the smearing.

The Width of the Smearing in eV	C_{11}	C_{33}	C_{44}	C_{66}	C_{12}	C_{13}	C_{16}	*B*	*G*	*E*
0.05 eV	481.0	451.7	268.6	260.2	149.5	129.2	−52.7	247.5	217.6	504.9
0.1 eV	483.7	452.7	271.0	261.8	148.9	130.7	−53.1	248.7	218.8	507.6
0.2 eV	483.7	453.5	270.4	261.1	148.9	130.6	−53.0	248.3	218.6	507.0

In order to verify the accuracy of the ideal strengths results from stress-strain method, the lowest ideal shear strength are calculated with the higher truncated energy of plane wave and the denser grid of the Brillouin zone integration. Here, we chose the (001)[100] slip system as the test direction. The calculated ideal shear strengths along (001)[100] direction with different truncated energy and *k* points are listed in Table S4.

Table S4. The convergence tests of cutoff energy and *k*-point mesh for ideal strength calculations.

	5 × 5 × 8	6 × 6 × 9	7 × 7 × 11
550 eV	27.5 GPa	27.5 GPa	27.5 GPa
650 eV	27.7 GPa	27.7 GPa	27.7 GPa
800 eV	27.3 GPa	27.3 GPa	27.3 GPa

Obviously, the higher truncated energy of plane wave and the denser grid of the Brillouin zone integration have only slight impact on the ideal shear strengths along (110)[001] direction, supporting that the calculations of ideal shear strengths in the present workare accurate and reliable. According to above analyses, we believe that the conclusion that the tetragonal $B_4CO_4$is not superhard can be trusted.

References

1. Zhang, Z.; Yang, Y.; Penev, E.S.; Yakobson, B.I. Elasticity, Flexibility, and Ideal Strength of Borophenes. *Adv. Funct. Mater.* **2017**, 1605059, doi:10.1002/adfm.201605059.
2. An, Q.; Goddard, W.A.; Cheng, T. Atomistic Explanation of Shear-Induced Amorphous Band Formation in Boron Carbide. *Phys. Rev. Lett.* **2014**, *113*, 095501.
3. Zhang, R.F.; Lin, Z.J.; Veprek, S. Anisotropic ideal strengths of superhard monoclinic and tetragonal carbon and their electronic origin. *Phys. Rev. B* **2011**, *83*, 155452.
4. Zhang, M.; Liu, H.; Li, Q.; Gao, B.; Wang, Y.; Li, H.; Chen, C.; Ma, Y. Superhard BC_3 in Cubic Diamond Structure. *Phys. Rev. Lett.* **2015**, *114*, 015502.
5. Zhang, R.F.; Lin, Z.J.; Zhao, Y.S.; Veprek, S. Superhard materials with low elastic moduli: Three-dimensional covalent bonding as the origin of superhardness in B_6O. *Phys. Rev. B* **2011**, *83*, 092101.
6. Zheng, B.; Zhang, M.; Chang, S.; Zhao, Y.; Luo, H.-G. Shear-Induced Structural Transformation for Tetragonal BC_4. *J. Phys. Chem. C* **2016**, *120*, 581–586.
7. Zhang, M.; Yan, H.; Zheng, B.; Wei, Q. Influences of carbon concentration on crystal structures and ideal strengths of B_2C_xO compounds in the B-C-O system. *Sci. Rep.* **2015**, *5*, 15481.
